# Association of Sarcopenia With Short-Term Outcomes After Liver Resection for Malignant Tumors

**DOI:** 10.7759/cureus.116001

**Published:** 2026-09-09

**Authors:** Sabuj Kumar Patra, Parijat Biswas, Md Abdullah-Hel Azom, Md Abdulla Al Mansur, Muhammad Salauddin, Amit Chowdhury, Emran Ali, Kamrul A Hillol, Murshidul Arefin, Mohammad Saief Uddin

**Affiliations:** 1 Department of Surgery, Directorate General of Health Services, Dhaka, BGD; 2 Department of Physiology, Green Life Medical College, Dhaka, BGD; 3 Department of Hepatobiliary, Pancreatic and Liver Transplant Surgery, Bangladesh Medical University, Dhaka, BGD; 4 Department of Hepatobiliary Surgery, United Medical College Hospital, Dhaka, BGD

**Keywords:** hepatocellular carcinoma, liver resection, postoperative complications, sarcopenia, skeletal muscle index

## Abstract

Background: Sarcopenia, characterised by reduced skeletal muscle mass, is increasingly recognised as a determinant of postoperative morbidity in patients with malignancy. Evidence regarding its association with short-term outcomes after liver resection for malignant tumours remains limited in Bangladesh.

Objective: This study determined the association between preoperative sarcopenia and short-term postoperative outcomes in patients undergoing liver resection for malignant tumours.

Methods: This prospective observational study was conducted in the Department of Hepatobiliary, Pancreatic and Liver Transplant Surgery, Bangladesh Medical University, from July 2024 to June 2025. Sarcopenia was assessed on preoperative non-contrast CT at the third lumbar vertebral (L3) level. Skeletal muscle area was normalised for height to calculate the L3 skeletal muscle index (SMI). Sex-specific SMI cutoffs of ≤52.4 cm²/m² for men and ≤38.5 cm²/m² for women were used. Demographic, perioperative, and short-term postoperative outcomes were compared between sarcopenic and non-sarcopenic groups.

Results: Twenty-seven patients were analysed; 15 (55.6%) were classified as sarcopenic and 12 (44.4%) as non-sarcopenic. The median postoperative hospital stay was longer in the sarcopenic group than in the non-sarcopenic group (14-16 vs. 7-9 days; Mann-Whitney U=180.0; rank-biserial correlation=1.00; p<0.001). Infectious complications occurred in 9/15 (60.0%) sarcopenic patients and 2/12 (16.7%) non-sarcopenic patients, corresponding to an OR of 7.50 (95% CI: 1.20-47.05; Fisher exact p=0.047). Operative time and intraoperative blood loss were not significantly different. No postoperative haemorrhage, bile leakage, liver failure, reoperation, 30-day readmission, or mortality was recorded.

Conclusion: Preoperative sarcopenia was common and was associated with prolonged hospitalisation and a higher incidence of postoperative infectious complications. Preoperative assessment of skeletal muscle status may identify patients who could benefit from nutritional optimisation and physical prehabilitation.

## Introduction

Liver cancer is a common malignancy with a poor prognosis, and global cancer statistics for 2020 identified liver cancer as the sixth most commonly diagnosed cancer and the third leading cause of cancer death, with approximately 906,000 new cases and 830,000 deaths [1]. Surgical resection remains a principal curative treatment for selected malignant liver tumours [2]. Although advances in surgical technique, anaesthesia, and perioperative care have reduced morbidity, clinically important postoperative complications continue to prolong hospitalisation, increase costs, and delay subsequent oncologic treatment [3,4]. Sarcopenia was originally introduced to describe the age-related decline in skeletal muscle mass and has since evolved into a broader clinical syndrome involving progressive loss of muscle quantity, strength, and function [5-9]. The European Working Group on Sarcopenia in Older People (EWGSOP) has emphasised the clinical consequences of impaired muscle status. Sarcopenia is also closely linked to malnutrition and cancer cachexia and has emerged as an adverse prognostic marker in patients with malignancy [5,10-14]. Previous studies have associated sarcopenia with postoperative complications, prolonged hospitalisation, impaired quality of life, and unfavourable long-term outcomes after liver and other major cancer operations [8,11,15,16]. However, local prospective data on the short-term consequences of sarcopenia after liver resection are limited. This study therefore aimed to determine the association between preoperative sarcopenia and short-term outcomes after liver resection for malignant tumours, with emphasis on postoperative infectious complications and length of hospital stay.

## Materials and methods

Study design and setting

This prospective observational study was conducted in the Department of Hepatobiliary, Pancreatic and Liver Transplant Surgery, Bangladesh Medical University (BMU), Dhaka, Bangladesh, from July 2024 to June 2025. The final analytic cohort comprised 27 adult patients undergoing liver resection for malignant tumours.

Study participants

Eligible patients were screened using the prespecified criteria summarised in Table 1. All participants underwent preoperative clinical assessment, biochemical investigations, and radiologic evaluation before surgery.

**Table 1:** Inclusion and exclusion criteria for study participants CT: computed tomography; L3: third lumbar vertebra.

Inclusion criteria	Exclusion criteria
Adult patients undergoing liver resection for a malignant tumour	Concomitant preoperative causes of muscle weakness, including major injury, fracture, or stroke
Preoperative CT suitable for skeletal muscle measurement at the L3 vertebral level	Diagnosed neuromuscular disorder
CT performed within 30 days before surgery	Missing CT data or CT images not extending to the L3 vertebral level

Sarcopenia was assessed from non-contrast axial CT images at the third lumbar vertebral (L3) level with radiologist support. Skeletal muscle was identified using a Hounsfield unit (HU) range of -29 to +150 HU. Cross-sectional skeletal muscle area was measured by manual tracing and normalised to height squared to obtain the L3 skeletal muscle index (SMI; cm²/m²) [17]. The formula is: SMI (cm²/m²) = Skeletal Muscle Area at L3 (cm²) / Height (m²).

Based on the criteria reported by Sreepriya et al. [18], sarcopenia was defined as an SMI ≤52.4 cm²/m² for men and ≤38.5 cm²/m² for women. Patients were categorised as sarcopenic or non-sarcopenic for analysis.

Data collection and outcome variables

Data were collected using a structured checklist by the investigator after written informed consent. Baseline variables included age, sex, body mass index, American Society of Anaesthesiologists (ASA) physical status [19], Eastern Cooperative Oncology Group (ECOG) performance status [20], comorbidities, serum albumin, serum bilirubin, blood glucose, and serum creatinine. Intraoperative variables included hepatic tissue texture, operative duration, and estimated blood loss. Postoperative outcomes included infectious complications, postoperative haemorrhage, bile leakage, postoperative liver failure, reoperation, length of hospital stay, 30-day readmission, and mortality. “Comorbidity present” represents the presence of at least one of the conditions listed: diabetes mellitus, hypertension, asthma or chronic obstructive pulmonary disease, chronic kidney disease, or hypothyroidism. Liver texture was assessed intraoperatively by the operating surgeon and recorded as soft or firm based on gross tactile assessment. Because this was a subjective clinical assessment rather than a standardised histological measurement, it should be interpreted as an exploratory operative characteristic. This exploratory prospective observational study did not have a single prospectively designated primary endpoint. The principal outcomes of interest were postoperative infectious complications and postoperative length of hospital stay; the remaining postoperative outcomes were considered exploratory secondary outcomes. The outcomes were measured within 30 days.

Statistical analysis

Primary statistical analyses were performed using IBM SPSS Statistics for Windows, version 26.0 (IBM Corp., Armonk, New York, United States; 2019). Additional verification of ORs, 95% CIs, and non-parametric effect-size estimates was performed using Python version 3.11 with SciPy version 1.16. Continuous variables were summarised as medians with interquartile ranges because of non-normal distributions and were compared using the Mann-Whitney U test. Rank-biserial correlation was reported as an effect size for the principal continuous outcome. Categorical variables were summarised as frequencies and percentages. Pearson chi-square tests were used when expected cell counts were adequate; degrees of freedom and Cramer's V were reported for chi-square analyses. Fisher's exact tests were used for sparse 2×2 tables. For binary associations, ORs with 95% CIs were reported. For 2×2 tables containing a single zero cell, the Haldane-Anscombe 0.5 continuity correction was applied to obtain a finite OR and approximate 95% CI; when both groups had zero events, the OR was not estimable. All tests were two-sided, and p<0.05 was considered statistically significant.

Ethical considerations

Ethical approval was obtained from the Institutional Review Board (IRB) of Bangabandhu Sheikh Mujib Medical University, now Bangladesh Medical University (registration no. 5181; reference no. BSMMU/2024/7587; approval dated 17 August 2024). The aims and procedures were explained in the local language, written informed consent was obtained, and confidentiality and anonymity were maintained. Declining participation did not affect the standard of clinical care.

## Results

Sarcopenia prevalence and baseline characteristics

Among the 27 analysed patients, 15 (55.6%) were classified as sarcopenic and 12 (44.4%) as non-sarcopenic (Figure 1).

**Figure 1:**
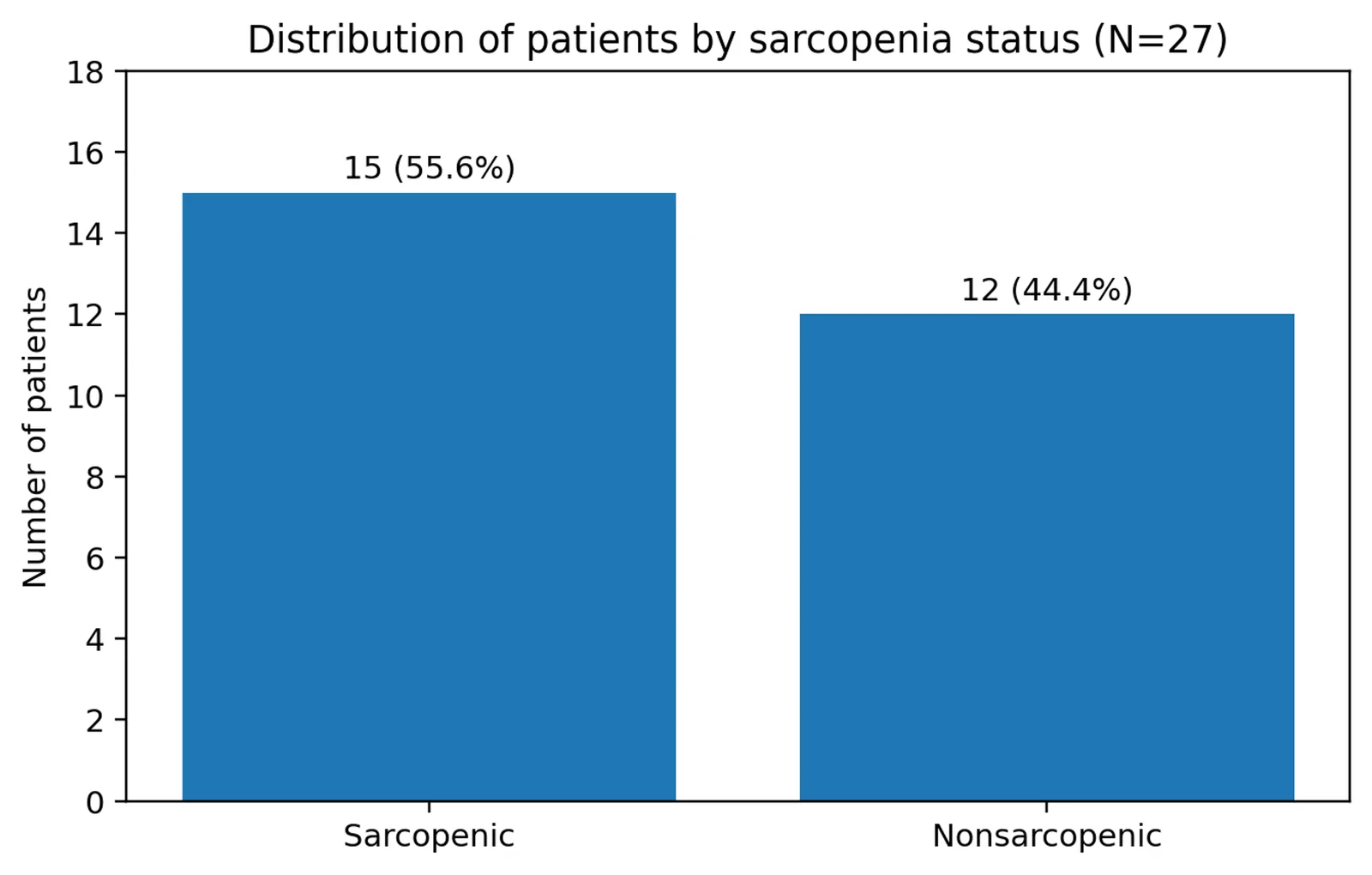
Distribution of patients by sarcopenia status (N=27) N: total number of patients.

The study identified sarcopenia in 9/12 (75.0%) men and 6/15 (40.0%) women (Table 2).

**Table 2:** Distribution of patients by skeletal muscle index at the third lumbar vertebral level (N=27) Skeletal muscle index is expressed in square centimetres per square meter. N: total number of patients; n: number of patients within the sex category.

Sex/skeletal muscle index threshold	Frequency	Percentage
Men (n=12): ≤52.4 cm²/m²	9	75.0%
Men (n=12): >52.4 cm²/m²	3	25.0%
Women (n=15): ≤38.5 cm²/m²	6	40.0%
Women (n=15): >38.5 cm²/m²	9	60.0%

Baseline characteristics were generally comparable between groups. Male sex was more frequent in the sarcopenic group (60.0% vs. 25.0%); the association did not reach statistical significance (Pearson χ²(1)=3.307, Cramer's V=0.350, OR=4.50, 95% CI: 0.85-23.80; p=0.069). Comorbidity was present in 53.3% and 25.0% of the sarcopenic and non-sarcopenic groups, respectively (OR=3.43, 95% CI: 0.66-17.93; Fisher exact p=0.239). Other baseline variables were not significantly different (Table 3).

**Table 3:** Baseline characteristics according to sarcopenia status (N=27) Categorical comorbidities included diabetes mellitus, hypertension, asthma, chronic obstructive pulmonary disease, chronic kidney disease, and hypothyroidism. Continuous values are median (interquartile range). CI: confidence interval; N: total number of patients; n: number of patients in each group; OR: odds ratio.

Variable	Sarcopenic (n=15)	Non-sarcopenic (n=12)	Effect estimate	p-value
Age, years	40.0 (35.0-56.0)	48.5 (38.5-55.0)	Mann-Whitney U test	0.478
Male sex	9 (60.0%)	3 (25.0%)	χ²(1)=3.307; Cramer's V=0.350; OR=4.50 (95% CI: 0.85-23.80)	0.069
Female sex	6 (40.0%)	9 (75.0%)	Reference	—
Body mass index, kg/m²	23.1 (22.5-25.5)	24.8 (22.7-26.8)	Mann-Whitney U test	0.241
Comorbidity present	8 (53.3%)	3 (25.0%)	OR=3.43 (95% CI: 0.66-17.93); Fisher's exact test	0.239
Comorbidity absent	7 (46.7%)	9 (75.0%)	Reference	—
Serum albumin, g/L	35.0 (33.0-40.0)	41.0 (33.0-42.7)	Mann-Whitney U test	0.403
Serum bilirubin	0.9 (0.5-1.2)	0.4 (0.3-0.6)	Mann-Whitney U test	0.095
Blood glucose, mmol/L	5.6 (5.3-6.0)	5.9 (5.4-6.1)	Mann-Whitney U test	0.351
Serum creatinine	0.9 (0.6-1.1)	0.9 (0.7-1.1)	Mann-Whitney U test	0.366

Intraoperative characteristics

Hepatic tissue texture was identical in distribution between the groups: firm texture was present in 5/15 (33.3%) sarcopenic and 4/12 (33.3%) non-sarcopenic patients (Pearson χ²(1)=0.000, Cramer's V=0.000, OR=1.00, 95% CI: 0.20-5.00; p=1.000). Operative duration and estimated blood loss were also comparable (Table 4).

**Table 4:** Intraoperative characteristics according to sarcopenia status (N=27) Continuous values are median (interquartile range). CI: confidence interval; N: total number of patients; n: number of patients in each group; OR: odds ratio.

Variable	Sarcopenic (n=15)	Non-sarcopenic (n=12)	Effect estimate	p-value
Soft hepatic texture	10 (66.7%)	8 (66.7%)	χ²(1)=0.000; Cramer's V=0.000; OR=1.00 (95% CI: 0.20-5.00)	1.000
Firm hepatic texture	5 (33.3%)	4 (33.3%)	—	—
Operative time, minutes	180.0 (150.0-180.0)	180.0 (150.0-180.0)	Mann-Whitney U test	0.836
Estimated blood loss, mL	150.0 (100.0-200.0)	175.0 (92.5-237.5)	Mann-Whitney U test	0.726

Median postoperative hospital stay was 15 (14-16) days in the sarcopenic group and 8 (7-9) days in the non-sarcopenic group (Mann-Whitney U=180.0; rank-biserial correlation=1.00; p<0.001) (Figure 2).

**Figure 2:**
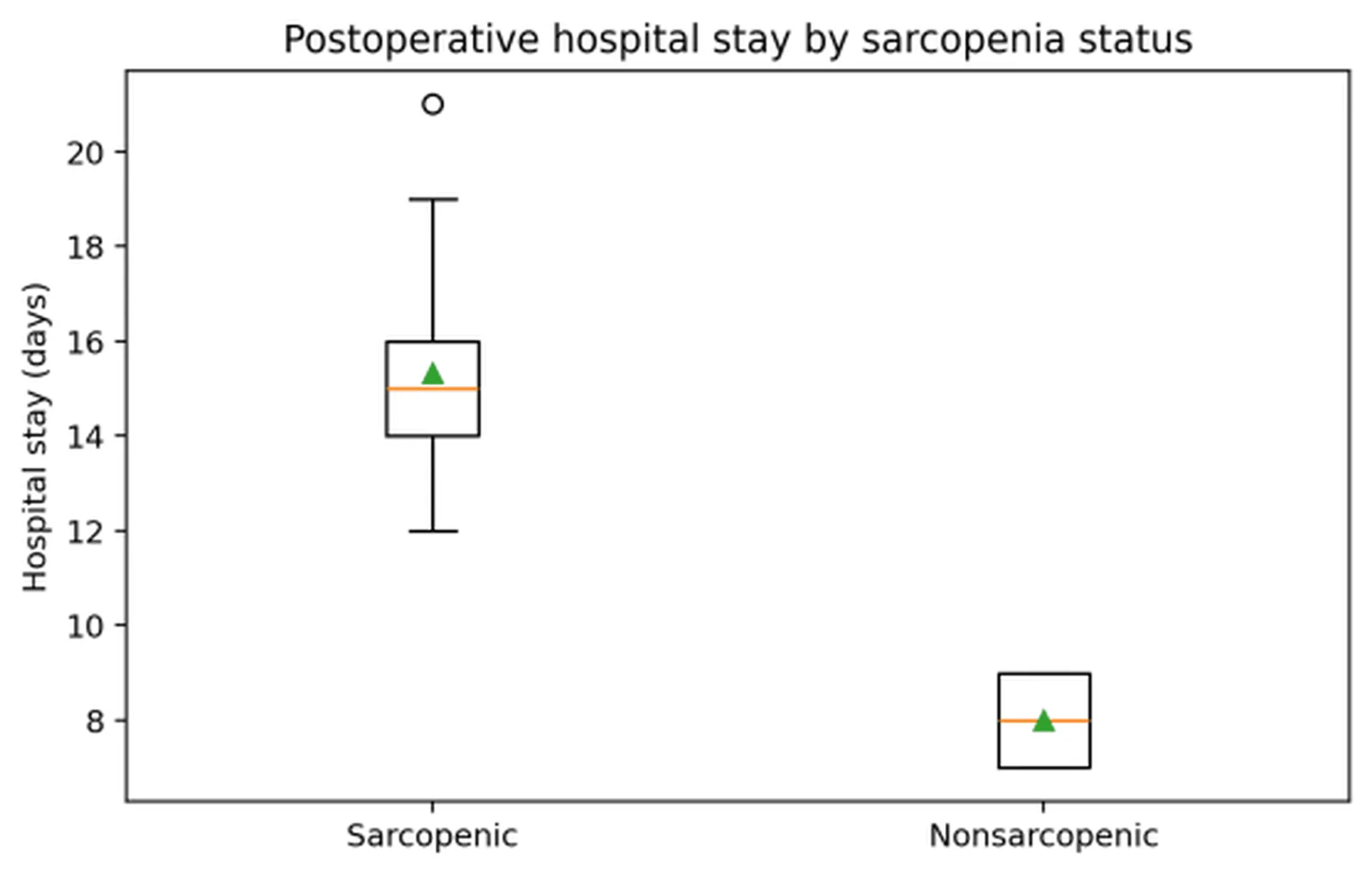
Postoperative hospital stay according to sarcopenia status (N=27) The box plot shows the median and interquartile range; whiskers represent the non-outlier range, and the marker indicates the mean. N: total number of patients.

Any infectious complication occurred in 9/15 (60.0%) sarcopenic patients compared with 2/12 (16.7%) non-sarcopenic patients, corresponding to OR=7.50 (95% CI: 1.20-47.05; Fisher exact p=0.047). For individual infectious outcomes, the ORs were 5.50 (95% CI: 0.55-55.50; p=0.182) for superficial surgical site infection, 4.63 (95% CI: 0.20-106.15; p=0.487) for deep incisional surgical site infection, 2.59 (95% CI: 0.10-69.34; p=1.000) for organ space infection, 0.79 (95% CI: 0.04-14.03; p=1.000) for urinary tract infection, and 2.59 (95% CI: 0.10-69.34; p=1.000) for pneumonia. The latter four estimates with a zero cell were calculated using the prespecified 0.5 continuity correction. None of the individual infection subtypes reached statistical significance, and their wide CIs reflect the small number of events. No patient in either group developed postoperative haemorrhage, bile leakage, postoperative liver failure, required reoperation, was readmitted within 30 days, or died; therefore, ORs for these outcomes were not estimable (Table 5).

**Table 5:** Postoperative outcomes according to sarcopenia status (N=27) Hospital stay values are presented as median (IQR) and compared using the Mann-Whitney U test. Fisher's exact tests were used for categorical outcomes. *For 2×2 tables with a single zero cell, the Haldane-Anscombe 0.5 continuity correction was used to calculate a finite OR and approximate 95% CI. CI: confidence interval; IQR: interquartile range; N: total number of patients; n: number of patients in each group; OR: odds ratio.

Outcome	Sarcopenic (n=15)	Non-sarcopenic (n=12)	OR (95% CI)	p-value
Postoperative hospital stay, days	15 (14-16)	8 (7-9)	—	<0.001
Any infectious complication	9 (60.0%)	2 (16.7%)	7.50 (1.20-47.05)	0.047
Superficial surgical site infection	5 (33.3%)	1 (8.3%)	5.50 (0.55-55.50)	0.182
Deep incisional surgical site infection	2 (13.3%)	0 (0.0%)	4.63 (0.20-106.15)*	0.487
Organ space infection	1 (6.7%)	0 (0.0%)	2.59 (0.10-69.34)*	1.000
Urinary tract infection	1 (6.7%)	1 (8.3%)	0.79 (0.04-14.03)	1.000
Pneumonia	1 (6.7%)	0 (0.0%)	2.59 (0.10-69.34)*	1.000

## Discussion

This prospective observational study found that preoperative sarcopenia was common among patients undergoing liver resection for malignant tumours and was associated with two clinically important short-term outcomes: prolonged postoperative hospitalisation and a higher incidence of infectious complications. The overall association with infectious complications was substantial (OR=7.50, 95% CI: 1.20-47.05), although the wide CI indicates considerable imprecision related to the small sample size. Analysis of specific infectious outcomes showed elevated point estimates for superficial surgical site infection (OR=5.50), deep incisional surgical site infection (OR=4.63), organ space infection (OR=2.59), and pneumonia (OR=2.59), whereas urinary tract infection showed no evidence of increased odds (OR=0.79). None of the subtype-specific associations was statistically significant, and these estimates should be interpreted as exploratory.

Sarcopenia was present in 55.6% of the cohort, a prevalence that is broadly consistent with the high rates reported in hepatobiliary oncology populations, although published estimates vary according to tumour type, ethnicity, imaging method, and the skeletal muscle index cutoff used [5,21,22]. Sarcopenia was more frequent among men in the present cohort, but the sex association did not reach statistical significance. Prior studies have also reported sex-related differences in sarcopenia prevalence [5,8,23].

The markedly longer hospital stay among sarcopenic patients is consistent with reports by Berardi et al. and Yang et al., who described worse short-term recovery after liver resection in patients with impaired body composition [8,23]. Other studies have not uniformly demonstrated an association with length of stay [24-26], which may reflect differences in sarcopenia definitions, extent of hepatic resection, perioperative pathways, and discharge practices. In the present study, operative time and blood loss were comparable between groups, making it less likely that the longer hospitalisation simply reflected a more difficult operation.

The higher frequency of infectious complications is biologically plausible. Skeletal muscle contributes to metabolic homeostasis and produces myokines that influence immune regulation. Sarcopenia also commonly coexists with inadequate protein reserves and impaired nutritional status, which may adversely affect immune cell function, tissue repair, and wound healing [27]. In the present cohort, superficial surgical site infection showed the largest subtype-specific point estimate (OR=5.50, 95% CI: 0.55-55.50), followed by deep incisional surgical site infection (OR=4.63, 95% CI: 0.20-106.15). Organ space infection and pneumonia each had corrected ORs of 2.59, while urinary tract infection had an OR of 0.79. However, all subtype-specific CIs were very wide and crossed 1.0, and none of the subtype-specific Fisher exact tests was significant. Accordingly, the statistically supported finding is the association with any infectious complication rather than with a particular infection subtype. This pattern is consistent with previous evidence linking low skeletal muscle mass to postoperative morbidity [8,11], but larger studies are required to determine whether sarcopenia preferentially increases the risk of specific postoperative infections.

Limitations

Several important limitations should be considered. First, this was a small, single-centre study involving 27 patients, resulting in limited statistical power and wide CIs, particularly for infrequent postoperative events. Second, the number of infectious events was insufficient to support a reliable multivariable model incorporating multiple clinically important covariates. Consequently, the reported associations are unadjusted and should not be interpreted as demonstrating that sarcopenia is an independent predictor of postoperative outcomes. Third, detailed information on tumour number, tumour size, tumour type, extent of liver resection, operative approach, background liver disease, and perioperative systemic therapy was not systematically collected. These factors may influence both skeletal muscle status and postoperative outcomes and therefore represent potential residual confounding. Fourth, sarcopenia was defined using CT-derived skeletal muscle mass without concurrent assessment of muscle strength or physical performance. Fifth, formal interobserver/intraobserver reliability testing of CT segmentation was not performed. Sixth, only short-term postoperative outcomes were evaluated, and oncological recurrence and long-term survival were outside the scope of the study. Seventh, formal standardised classifications were not incorporated prospectively into the original data collection. We have therefore added the operational definitions used clinically and acknowledged the absence of prospectively applied standardised definitions as a limitation. Finally, detailed tumour type information was not systematically available; therefore, potential differences in tumour biology and disease-specific surgical complexity could not be evaluated.

Clinical applicability

These findings support incorporating a simple review of preoperative CT-derived skeletal muscle status into routine risk assessment for patients being considered for liver resection. Patients identified as sarcopenic could be referred for focused nutritional assessment, optimisation of protein and energy intake, and individualised exercise or resistance training programs before surgery when oncologic timing permits. Sarcopenia status may also inform counselling regarding postoperative recovery, closer wound and infection surveillance, early mobilisation, and proactive discharge planning. Because this study demonstrates association rather than causality, these interventions should be considered. A practical implication is that preoperative CT scans already obtained for oncologic assessment can also be reviewed for low skeletal muscle mass to identify higher-risk patients. When oncologic timing permits, these patients may be referred for formal nutritional assessment, optimisation of protein and energy intake, individualised exercise or resistance-based prehabilitation, early mobilisation, closer postoperative wound/infection surveillance, and proactive discharge planning.

## Conclusions

In this small single-centre prospective cohort, CT-defined sarcopenia was common among patients undergoing liver resection for malignant tumours and was associated with longer postoperative hospital stay and a higher frequency of infectious complications. Because the estimates were derived from a small sample and were not adjusted for important tumour-, liver-, and procedure-related factors, these findings should be considered preliminary and hypothesis-generating. Larger, adequately powered multicentre studies incorporating detailed tumour characteristics, extent of hepatic resection, background liver status, standardised complication definitions, and multivariable adjustment are required to determine whether sarcopenia independently predicts postoperative outcomes and whether targeted prehabilitation can improve recovery.
